# It's not the '*what*', but the '*how*': Exploring the role of debt in natural resource (un)sustainability

**DOI:** 10.1371/journal.pone.0201141

**Published:** 2018-07-20

**Authors:** Julen Gonzalez-Redin, J. Gareth Polhill, Terence P. Dawson, Rosemary Hill, Iain J. Gordon

**Affiliations:** 1 Information and Computational Sciences, James Hutton Institute (JHI), Aberdeen, Scotland, United Kingdom; 2 Geography and Environmental Science, School of Social Sciences, University of Dundee (UoD), Dundee, Scotland, United Kingdom; 3 Department of Geography, King’s College London (KCL), Strand, London, England, United Kingdom; 4 CSIRO Land and Water, Division of Tropical Environments and Societies, James Cook University (JCU), Cairns, Queensland, Australia; 5 Tropical Environments and Societies, James Cook University (JCU), Townsville, Queensland, Australia; 6 Fenner School of Environment & Society, Australian National University (ANU), Canberra, Australian Capital Territory, Australia; Universidad de Castilla-La Mancha, SPAIN

## Abstract

A debt-based economy cannot survive without economic growth. However, if private debt consistently grows faster than GDP, the consequences are financial crises and the current unprecedented level of global debt. This policy dilemma is aggravated by the lack of analyses factoring the impact of debt-growth cycles on the environment. What is really the relationship between debt and natural resource sustainability, and what is the role of debt in decoupling economic growth from natural resource availability? Here we present a conceptual Agent-Based Model (ABM) that integrates an environmental system into an ABM representation of Steve Keen’s debt-based economic models. Our model explores the extent to which debt-driven processes, within debt-based economies, enhance the decoupling between economic growth and the availability of natural resources. Interestingly, environmental and economic collapse in our model are not caused by debt growth, or the debt-based nature of the economic system itself (i.e. the ‘*what*’), but rather, these are due to the inappropriate use of debt by private actors (i.e. the ‘*how*’). Firms inappropriately use bank credits for speculative goals–rather than production-oriented ones–and for exponentially increasing rates of technological development. This context creates temporal mismatches between natural resource growth and firms’ resource extraction rates, as well as between economic growth and the capacity of the government to effectively implement natural resource conservation policies. This paper discusses the extent to which economic growth and the availability of natural resources can be re-coupled through a more sustainable use of debt, for instance by shifting mainstream banking forces to partially support environmental conservation as well as economic growth.

## Introduction

Humanity has failed to make sufficient progress in solving most environmental challenges, such as climate change, freshwater availability, deforestation, marine fisheries collapses, among others [1]. This has produced a number of discussions that highlight the impossibility of continuous economic growth within the ecological boundaries of our planet [2, 3]. Therefore, preventing the collapse of the systems that support life on this planet will probably require economic growth to be decoupled from the environmental impact of the economy [4].

A popular critique of the economic-financial system says that, because banks create money in the form of interest-bearing debt, the system necessarily requires an expanding money supply to pay this interest [5]. The expanding money supply is argued to enhance an economic growth imperative that forces society to generate an ever-increasing income flow. As a result, more and more debt is accumulated, while more future growth is needed to repay the debt [6]. Thus, the cycle continues. This monetary business-as-usual trajectory requires the production of more goods and services [7]–along with pollution and resource use–and enhances the probability of system breakdown [8].

In this regard, the last financial crisis in 2008 confirmed that the dominant neoclassical models of macroeconomics were seriously flawed [9]. Policy makers, who relied upon models that were not able to predict the actual behaviour of financial markets, were misled, and the credibility of economic theory has been widely called into question [10]. Hence, there is a need to develop new economic models that replicate the actual nature of the economy [9] and transdisciplinary approaches that address the impact of the economy on natural systems [11, 12]. While there has been much attention on studying the actual nature of both economic and ecological systems independently, the attempts to do so for coupled social-ecological systems (SES) are at an early stage [13]. SESs are dynamically complex systems composed of people and nature [14], emphasizing that humans should be seen as a part of, not apart from, nature [15]. Modelling and exploring coupled SESs is an important step forward, since those economic models not considering environmental implications (e.g. resource availability, pollution) are more likely to show pathways towards false sustainable economic states [16]. Yet, the capture of environment constraints, through integration of environmental variables within economic models, could help developing more realistic, long-run scenarios [17].

In this regard, as ecology and economics are concerned with interactions among individuals and entities, both have much to gain from computer modelling tools for complex systems, including Agent-Based Modelling (ABM). ABM simulates systems of autonomous and heterogeneous agents, which interact with each other and their environment, making decisions and changing their actions and the environment as a result of these interactions [18]. ABMs are argued to be helpful for studying complex dynamics in SESs [19, 20], as well as gaining insights that support the sustainable management of natural resources [21]. This paper presents a conceptual ABM that examines the relationship between debt and natural resource (un)sustainability. The model is used to answer the following research question: what is the impact of debt-driven economic growth on natural resource dynamics in complex coupled SES? The novelty of this research question lies in the fragmented and circumstantial evidence regarding the relationship between debt dynamics and sustainability [22]. In particular, our model focuses on debt dynamics derived from credit borrowing by firms from banks, i.e. not considering household and government debt. We hypothesise that high-debt dependent production systems exert negative effects on the capacity of the economic system to enhance the sustainable use of natural resource stocks. To test this hypothesis, our SES is composed of a simple environmental-resource system within an ABM inspired by Steve Keen’s pure economic models [9, 23]. The novelty and relevance of integrating an environmental system into Keen’s economic models is based on these models solving the paradox of how monetary profits can be generated by private actors in debt-based economies. As explained in the methodology, we argue that this idea justifies a continuous, yet potentially unsustainable, economic growth. However, Keen’s models do not consider the impacts that debt-growth cycles have on the environment, which in our view provides a suitable platform to explore the relationship between debt and natural resource (un)sustainability. The next sections describe the modelling framework in detail, followed by model findings and a discussion on the extent to which monetary debt is a key factor driving natural resource (un)sustainability in complex SES.

## Methodology

### Agent-based social-ecological systems modelling: Integrating an environmental system into Steve Keen’s economic models

The lack of complexity in neoclassical economic models reduces their capacity to describe, in detail, any society ever observed [24]. For instance, scholars argue that the mainstream economic models used by some financial entities (e.g. Wall Street) have not been built to understand the complexities of the economic system, but rather to provide tractable results and straight-forward ways to implement policies [25]. Furthermore, while attempts to model the economic system exist, for instance through system dynamics modelling (e.g. [26]), most economic models only focus on financial processes and do not analyse their impacts on the environment. These models have been capable of modelling economic phenomena such as money [26], bounded rationality [27] or income distribution [28], yet they are especially weak in regard to ecological variables and to feedback channels between the environment and economy. Thus, the contribution of economic models that explore alternative structures for more sustainable economies, such as “green growth” [29], “steady state” or “degrowth” approaches [30, 31], is rare.

There is a need to understand complex systems behaviour, such as those between economic systems and the environment, through novel non-linear modelling tools. In this regard, ABM has been receiving significant attention recently, being widely employed across fields that are as diverse as biology [32], business [33], education [34], geography [35], health care [36], political sciences [37] and sociology [38]. ABM explores how interactions between agents (e.g. entities, people, non-human beings) affect the system and their own behaviour through the property of emergence [39]. The main characteristics of ABMs, and the benefits of using ABM to simulate complex SESs, include: (i) capturing of *emergent* system phenomena; (ii) agent *heterogeneity* (allowing modelling complex and non-linear behaviour); (iii) integration of socio-economic *networks* and physical space-based *interactions*; and (iv) *dynamical natural* descriptions of SESs, instead of solely analysing final model results [40].

These characteristics provide ABM the capacity to model complex SESs through interdisciplinary approaches. Yet, the majority of ABMs in literature are single-disciplinary. For instance, the field of Agent-Based Computational Economics (ACE) has explored features of economies as complex systems [26, 27], while Individual-Based Modelling (IBM) has been widely used in ecology, even to a greater extent than in economics [41, 42].

The present paper expands the ABM literature in complex coupled SES and economic-environmental dynamics. For that purpose, Steve Keen’s [9] economic model–which uses the so-called “Circuit Theory” as a framework (see [43])–is used as a basis to build the economic dimension of the ABM. Moreover, elements (mainly financial (Ponzi) speculation) from another of Keen’s models [23]–not included in [9]–are also integrated in the economic dimension of our ABM. Keen’s work is an alternative to traditional economic models that explicitly considers the role of money, debt and banks. In particular, Keen’s [9] model shows how money circulates among banks, firms and households under the current economic paradigm. The main interest in using Keen’s work lies in its capacity to explain the paradox of how monetary profits are generated in debt-driven capitalist systems–an explanation that economics had failed to provide satisfactorily until then [44]. In short, Keen’s models demonstrated how firms and banks can make profits, through credit borrowing and lending, regardless of their increasing debt burdens and the potential economic collapse that these processes may cause. This scenario justifies the profit-seeking behaviour of these actors, as well as never-ending economic growth, where firms prioritize debt-driven resource extraction processes because these provide them with short-term profits. This economic context, which we argue aligns with the business-as-usual paradigm of the current economic system, could be detrimental to achieving natural resource sustainability.

The model presented in this paper uses Keen’s debt-based approach to economic growth as a basis in order to build an ABM that explores the impacts of debt-based economies on natural resource sustainability. In particular, the model examines those debt-driven socio-economic factors that could be enhancing the decoupling between economic growth (i.e. GDP) and natural resource availability.The following section describes the ABM, based on a coupled SES that integrates a simple environmental system (and the corresponding economic-environmental feedbacks) within a debt-based economic system inspired by Keen’s models [9, 25].

### Model description: Overview, Design Concepts and Details (ODD)

The model was built using NetLogo as the modelling software [45]. Grimm *et al*.’s [46, 47] ODD (Overview, Design concepts and Details) model description protocol was used to give an overview of the model. Here the ‘Entities, state variables and scales’ and ‘Process overview and scheduling’ sections of the ODD are integrated and presented as one single section, while the rest of the protocol can be found in the S1 Appendix.

The model consists of agents interacting within three different markets, i.e. credits, goods and labour markets, as well as the environment. The environment consists of a grid of 100 × 100 land parcels (patches), each of them with a biomass (resource) stock. The different types of agents in the model include: firms–which use bank credits to finance production of goods (for which extracting natural resources is needed) that are then sold to households; a commercial bank–which lends credits (loans) to firms under different financial situations; speculators–which also borrow credits to bet on the goods (assets) produced by firms, but have no hand in the sale of such goods; and the government–which implements conservation policies to preserve the stock of natural resources and counterbalance the environmental impacts exerted by economic growth. Note that only debt dynamics originated by firms and speculators are considered, i.e. neither households nor the government borrows credits from the bank in our model. Fig A in S1 Appendix presents a Unified Modelling Language (UML) class diagram of the model, specifying and showing the links among model entities and parameters; Table A in S1 Appendix shows a description of the parameters modelled for each entity (i.e. agent type), as well as their initial values.

The following are the specific processes that take place every time step in the model. The functions and algorithms computed by these processes are displayed in Table 1 –see also the ODD section ‘Submodels’, in S1 Appendix, for a more detailed description of model functions and processes. Note that some functions are adapted from Keen [9, 23] to our particular modelling context, by disaggregating the equations and algorithms computed by homogeneous entities (in Keen’s models) to the heterogeneous nature of ABM. The model processes include: (i) patches compute biomass stock; (ii) firms extract resources; (iii) households compute demand, movement and energy input/output; (iv) firms compute prices and sales; (v) firms compute labour and finance; (vi) banks compute finance; (vii) firms borrow credits; (viii) firms consider business expansion; (ix) speculators compute speculation; (x) firms and speculators compute credit repayment; and (xi) government computes natural resource conservation policies.

**Table 1:** Main model functions and the corresponding algorithms.

	function name	acronym	algorithm
(1)	biocapacity	*B*	$B=R_{s}∙F_{y}∙F_{eq}$
(2)	resource extraction	*R*_*e*_	$R_{e}=(D∙L∙c)-B_{r}$
(3)	demand	*D*	$D=H_{c}/(P∙v)$
(4)	investment	*K*	$K=\left({AGD}_{t-1}∙L∙F_{c}∙M_{c}\right)$
(5)	price	*P*	$P=(D∙P_{k})/B_{r}$
(6)	productivity	*p*	$p=(F_{c(t)}-F_{c(t-1)})/L$
(7)	nominal wage	*W*_*n*_	$W_{n}={(W}_{c}∙F_{c})/L$
(8)	speculation	*P*_*k*_	$P_{k}=k\left(g\right)∙Y$

First, each land parcel (patch) computes one resource stock (*R*_*s*_), which increases over time following a resource growth function. Related to this, each patch computes its own biocapacity (*B*) (*function 1*, Table 1), which refers to the capacity of the land to produce useful biomass (i.e. resources with potential to be converted to production goods), and to absorb waste biomass generated by firms [48]. *B* varies based on *R*_*s*_, yield factor (*F*_*y*_) and equivalence factor (*F*_*eq*_); *F*_*y*_ accounts for differences between countries in productivity of a given land type, while *F*_*eq*_ converts a specific land type into a universal unit of biologically productive area [48]–note that our model uses the values for forest-land for both *F*_*y*_ and *F*_*eq*_, due to the similarity between the natural resource modelled (in terms of growth-rate and extraction process) and forest-land plantations. Firms extract resources from their current patch location through a resource extracting (*R*_*e*_) function (*function 2*). The amount of resources extracted by each firm varies with each time step based on demand (*D*) for goods (*function 3*), labour (*L*) (i.e. workforce), the amount of resources available in firms’ biomass reserve (*B*_*r*_) (which permits firms to cope with periods with excess of demand or lack of resource availability), and a resource conversion factor (*c*). Firms’ resource extraction processes have a monetary cost for them (*function 4*), related to the investment (*K*) needed, in each time step, to generate enough goods to meet the aggregate household demand (*AGD*), also considering *L*, the firm’s monetary capital (*F*_*c*_) and an extraction-demand correction mechanism (*M*_*c*_). Harvested resources are stored in each firm’s reserve, and then sold to households–after conversion to goods–at a specific price (*P*) value (*function 5)*; *P* varies upon *D*, *B*_*r*_, and a speculation rate (*P*_*k*_) (explained below); note that all firms in the model sell the same type of good (modelling different types of good will be subject of a future version of the model). *D*in our model changes based on *P*, households’ monetary capital (*H*_*c*_), an accelerator effect (*v*), and distance–note that (*v*) is related to the GDP, where an increase in GDP enhances (*F*_*c*_) investment spending in resource extraction. Productivity (*p*) (*function 6*) states the effectiveness of firms’ productive effort, which varies depending upon each firm’s profits from one year to the next (*F*_*c(t)*_*−F*_*c(t-1*)_) and *L*. Households work for firms and receive a nominal wage (*N*_*w*_) following *function 7*.

With regard to the bank, it possesses two different monetary capital stocks–withdrawable capital and bank reserves; while the bank reserve stock holds the monetary capital designated to lend credits to firms, withdrawable capital retains household deposits available for direct withdrawal for consumption of goods. The bank lends credits to firms based on each firm’s particular financial situation, and firms have to pay the debt (with interests) back to the bank. The bank also pays deposit interests to households. Thus, the bank’s net profits vary based on the surplus generated from the difference between household deposits (losses) and credit interests from firms and speculators (gains)–note that households do not borrow credits in our model, thus not influencing debt dynamics. Credits are used by firms to cover different expenses, i.e. resource extraction processes, wages, investments in improving technological efficiency, and equipment and materials–note that technological efficiency is only applied to the resource extraction processes, i.e. to increase the productivity of extracting resources. Similarly, firms may use credits to fund business expansion, based on creating one new branch/firm in an area with high resource availability. The monetary capital available from the bank for credit lending varies based on the type of economic/banking system modelled. Thus, two different systems are computed: fractional-reserve banking system–with high capital available for credit lending–and full-reserve banking–with limited capital (see ‘Scenario rationale’ below).

Furthermore, speculators also borrow credits from the bank in order to carry out speculative processes (*P*_*k*_), based on purchasing derivatives through *function 8*, i.e. instruments to bet on what price the produced good (i.e. asset) will reach by a future date. Speculation increases with further economic growth rate (*k*_*g*_) and model output (*Y*), i.e. amount of goods producer per time step. Speculators have no hand in the sale of goods, i.e. they are not the buyer or the seller, yet they are able to affect prices through inflationary and deflationary processes. Both speculators and firms repay credits, with interests, to the bank. Finally, the government implements different policies to enhance conservation of resources when the system’s natural resource stocks drop below specific thresholds (see ‘Scenario rationale’ below).

**Fig 1:**
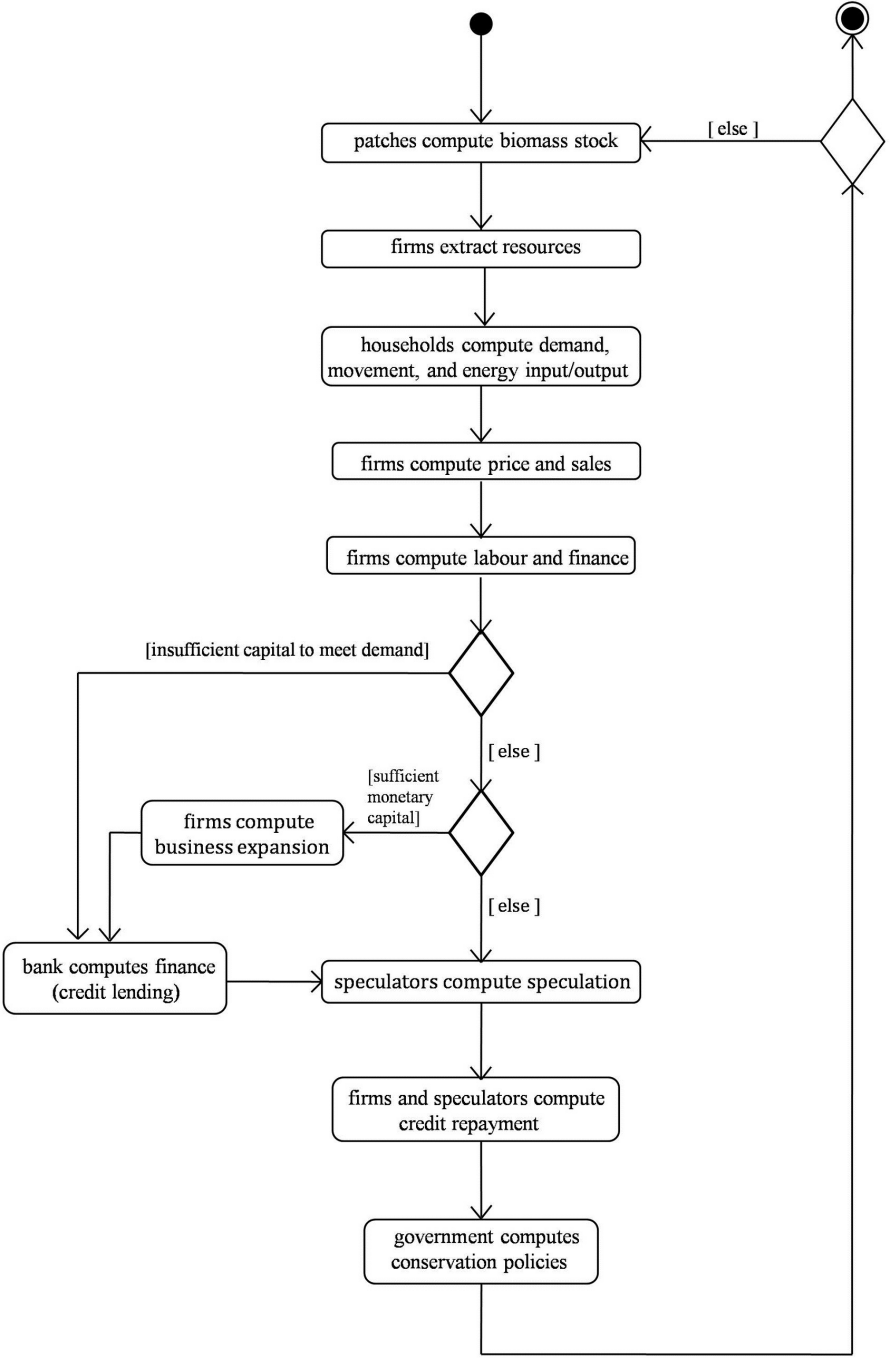
UML activity diagram. Structure diagram for each time-step in the model, showing the step by step processes computed by agents and patches.

Fig 1 shows a UML activity diagram of the model. This shows the links among the above-noted processes and the order in which these processes occur in each time step.

### Scenario rationale, sensitivity analysis and model calibration

The model simulates two scenarios; namely fractional-reserve and full-reserve banking systems. The fractional-reserve computes a cash reserve ratio of 0.02 –following the European Union’s reserve [49]. Cash reserve ratios set the minimum amount of reserves (i.e. the bank’s holding of deposits that are not lent out as credits) that must be held by the bank. Thus, 2% is the amount of households’ deposits available for withdrawal from banks (i.e. withdrawable capital, for consumption of goods) under fractional systems, while 98% is available solely for credit allocation to firms (i.e. bank reserves). By contrast, the full-reserve banking system computes a cash reserve ratio of 1, where the amount of capital available for credit borrowing is very limited, since the bank must keep 100% of households’ deposits available for withdrawal. Due to the gains that the bank makes from the difference between credit interest (gains) and deposit interest (losses)–where the former are normally higher than the latter–the bank, under full-reserve systems, normally allocates more than 0% of capital for credit lending.

Computing both debt-based (i.e. fractional) and non-(or limited) debt-based economic systems allows the comparison of the role of debt in the economy and its impacts on natural resource sustainability. Moreover, the fractional-reserve system scenario computes various sub-scenarios; these are based on government intervention in the economy through the implementation of conservation policies, which help counterbalancing the negative effects exerted by economic growth on natural resources. Thus, the government in our model enhances natural resource conservation when the total stock of natural resources in the system drops below specific thresholds, provided by the parameter *critical-biomass-stock* (explained below). More specifically, the policies implemented by the government are focused on (i) forcing firms to decrease investments in technological development to improve production efficiency (i.e. implementation of the precautionary principle); (ii) limiting speculation on assets and speculative artificial markets; (iii) enlarging the protected area network by decreasing the number of patches available for resource extraction; and (iv) forcing firms to restore the land used for resource extraction processes once the natural resources stock is depleted. Note that no government intervention is computed under full reserve system scenarios, due to the very limited impacts exerted on the environment by the economy in this scenario–almost non-existent compared to fractional reserve systems.

An OFAT (One-factor-at-a-time) sensitivity analysis was performed [50]. The sensitivity analysis consisted of observing changes in agents’ behaviour, as well as in model outputs, with all except one of the parameters held constant. Due to the model being particularly sensitive to changes in the *critical-biomass-stock* parameter, this variable was varied through a series of different values. This parameter states different natural resource threshold values, where the government starts implementing conservation policies (i.e. government intervention) if the total stock of natural resources in the system drops below predefined values for *critical-biomass-stock*. Thus, the sensitivity analysis performed–see S2 Appendix–shows the extent to which the main environmental (i.e. ‘Natural resource stock’) and economic (i.e. ‘Real GDP growth’) indicators are affected under different values of this parameter. Each *critical-biomass-stock* value selected for the analysis was run 100 times, which is considered a reasonable number of runs to generate valid and stable predictions in stochastic simulations [51]. The average and standard error values from all the runs regarding the indicators selected are shown in the result figures.

Model calibration–see S3 Appendix–followed a comparative analysis between our model’s and Keen’s [9, 23] results, where the objective was to assess as to whether our model was able to reproduce similar patterns to those from Keen’s models. Among the scenarios modelled, the results from the fractional-reserve system (with no government intervention) were used for the calibration process. This is because Keen’s models are based on pure debt-based macroeconomic systems, with no full-reserve system included. Furthermore, government intervention in Keen’s models do not have the same objective as in our model; where the role of government in his model is to help overcoming an exogenously (to the model) set credit crunch, while our model seeks to explore the endogenous role of conservation governance in preserving natural resources. Regardless of the conceptual nature of our model, its qualitative behaviour shows matching patterns with regard to those from Keen [9, 23]–see S3 Appendix; especially during the first period of the simulation (i.e. economic boom) under our fractional-reserve system (see Results). After then, our results and Keen’s partially differ, mainly based on the integration of environmental constraints to our model, which contrast to Keen’s pure macroeconomic approach.

## Results

**Fig 2:**
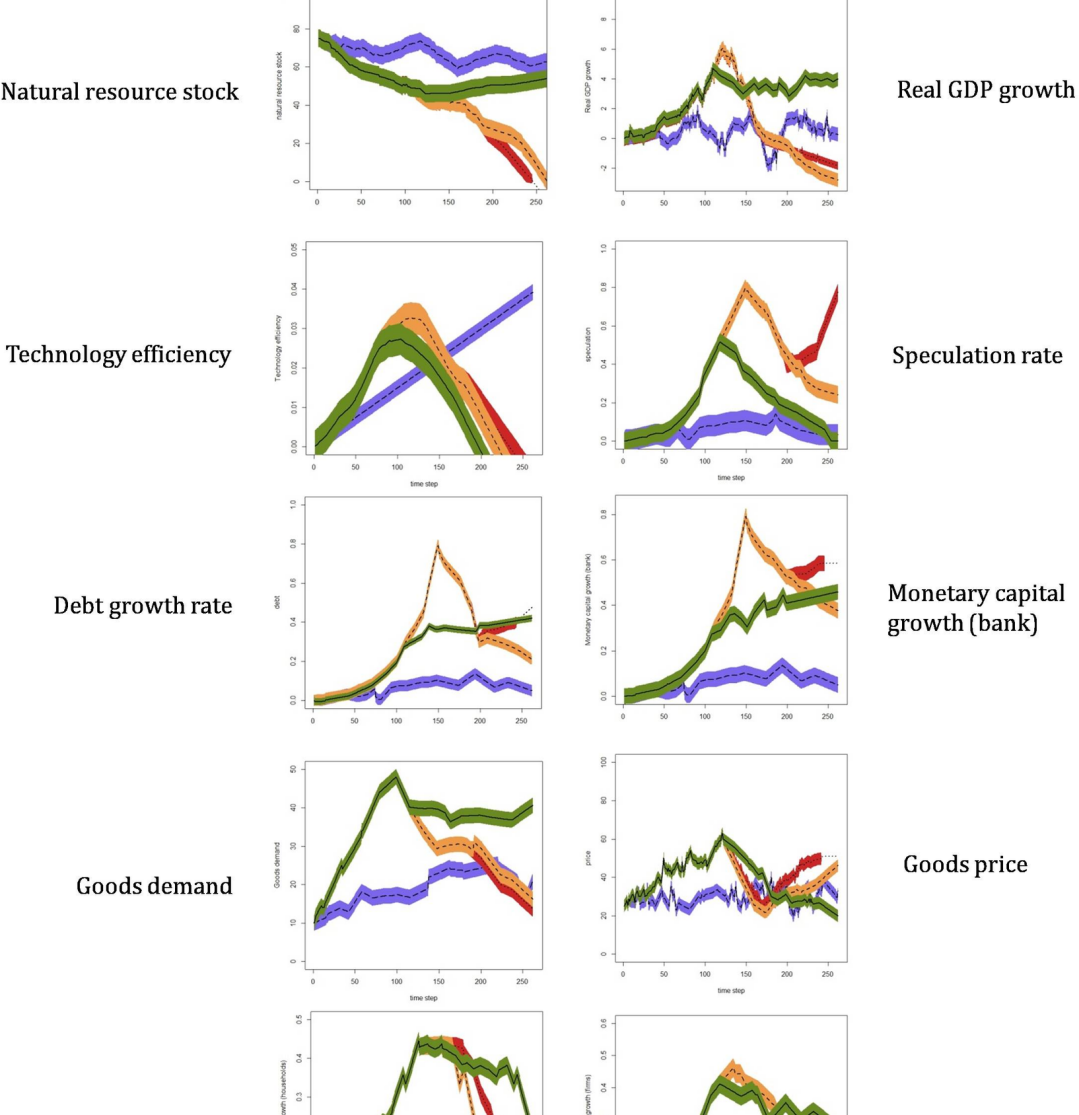
Simulation results. Results obtained for the indicators selected under a fractional-reserve system–without government intervention (red dotted line) and with government intervention when the total natural resource stock is at 25% (yellow short-dash) and 50% (green solid line)–and under a full-reserve system (purple long-dash line). Black coloured curves (i.e. dotted, solid, short and long-dashed) show the mean values, whereas coloured bands represent the standard error bars including all the runs computed for each indicator under every scenario.

The results analysis compares and identifies qualitative differences in trends among indicators. Fig 2 shows the modelling results obtained under non-debt (full-reserve) and debt-based (fractional-reserve) economic systems, the latter also including government intervention through conservation policies for two different *critical-biomass-stock* thresholds (25% and 50%). The selection of these two values–among a total of twelve–was based on the results obtained from the sensitivity analysis (see S2 Appendix).

### Fractional-reserve and full-reserve systems (no government intervention)

Under debt-based (fractional) economic systems, with no government intervention, firms are able to cover their daily expenditures, wages and investments for technological development, due to the high availability of natural resources and bank credits. In the processes of selling goods and borrowing (lending) credits, both firms and banks gain profits. Overall, this process maintains continuous economic growth, fuelled by loans that drive increasing labour productivity. The increasing level of debt, and the tendency to borrow more when profits increase (in anticipation of a rise in income and the promise of future wealth creation), has no apparent effect on the economy at this point (see the period 0–120 for ‘Real GDP growth’ and ‘Monetary capital’ indicators, red-dotted curve, Fig 2). From an environmental perspective, the increasing extraction of resources affects natural resource stocks, thus showing decreasing values during the simulation.

At the same time, the rise in speculation shows that some monetary capital funding economic growth enters the system according to speculative goals, instead of purely production-oriented goals. This is due to the presence of speculator agents, which also borrow credits to gain future profits by trading assets on a rising market. As credit borrowing by speculator agents occurs when prices and GDP increase, this process starts enhancing price inflation and, as a result, further speculation. This reinforcing cycle enhances a growing debt burden that adds no productivity value to the system(see the period 0–120 for ‘Debt growth rate’).

Once the simulation exceeds 100-time-steps, price inflation has reached its peak due to speculation, economic growth and the increasing debt burden. As a result, demand over goods and firms’ monetary capital decreases, which in turn reduces labour–due to the inability of firms to pay for households’ wages. This loss of purchasing power by households enhances a deflationary process, while firms are no longer able to fund investment in technological development for improving production efficiency. Furthermore, price deflation reduces speculation, since the number of speculators in the system is directly correlated with inflationary processes. Thus, most speculators go bankrupt, which reinforces further price deflation. In particular, bankruptcy takes place among those speculators with low monetary capital, who are not willing to borrow further credits. Because most speculators are not able to pay back debt credits to the bank, unpaid debt stocks become the bank’s debt. This reduces the capital available from the bank for credit lending, thus creating a domino effect affecting firms. From an environmental perspective, the reduction of resource extraction processes benefits natural resource stocks, which show steady state values for the first time during the simulation (see the period 125–175 for ‘Natural resources stock’ indicator).

Eventually, the reduction in prices encourages higher household demand for goods and a period of system stability. However, because this rise is not sufficient to increase firms’ monetary capital, GDP values continue to decrease–albeit at a lower rate than under high speculation values. The economy starts to recover slightly, and the rise in prices (> 175-time-steps) attracts speculator agents again, which enhances debt stocks and further increase in prices–albeit at a lower rate than at the beginning of the simulation. Because natural resources are almost fully depleted from the excessive resource extraction, both firms’ income and production of goods are affected, thus reducing the capacity of firms to repay borrowed credits back to the bank. This new context affects banks, firms and speculator agents negatively. Eventually, natural resource collapse occurs, thus creating the breakdown of the system and ending the simulation.

Fig 2 also shows the results obtained under a full-reserve system (see purple long-dashed curves in this figure), where the bank is forced to keep 100% of the deposits available for withdrawal. As previously explained, the amount of capital allocated by the bank for credit lending is not 0%–albeit very low, since the bank still generates money for credit lending from the difference between credit interests (gains) and deposit interests (losses).

Under this scenario, most environmental and economic indicators remain relatively stable over time, compared to those under fractional-reserve systems. Yet, this stability is achieved at low ranges of values regarding ‘Natural resources stock’, ‘Real GDP growth’, ‘Debt growth rate’, ‘Speculation rate’, and ‘Monetary capital growth (firms, households and bank)’, as well as the rest of indicators. Basically, the low allocation of credits (debt) by the bank for both production-oriented (through firms) and speculative (through speculators) goals creates a system with low income and profits, yet also with low environmental impacts. As a result, model results neither show economic nor environmental collapses during the simulation period, since the risk of natural resource depletion, as well as high speculation, debt or inflation rates (which increase the probability of economic collapses) is low.

### Government intervention in fractional-reserve systems

The implementation of government policies under a fractional-reserve system was modelled. In our model, conservation governance is used as a process to counterbalance the negative environmental impacts exerted by economic activities. The policies focus on enhancing the sustainability of natural resources only if their total stock in the system drops below two specific thresholds, i.e. 25% and 50% (of the initial stock). Rather than the specific time steps and natural resource threshold values at which conservation policies are implemented (i.e. tipping points), our analysis focuses on the importance of government intervention, as a whole, under potentially unsustainable debt-based economic systems.

Fig 2 shows that conservation policies implemented only after ‘Natural resources stock’ drops below 25% of its initial capacity are not able to prevent system collapse (see yellow short-dash curves). In particular, the small amount of natural resources left by then, as well as the high rates of technological development and resource extraction processes, create an unsolvable context for the government in terms of avoiding system collapse. Interestingly, GDP, after government intervention, decreases over time at a higher rate than under fractional-reserve systems with no government intervention. In contrast, conservation policies implemented before the system’s total natural resource stock drops below 50% (green solid curves) are able to enhance natural resource stability over time, with no system collapses during the simulation period.

## Discussion

The robustness of our model results is based on the integration of Steve Keen’s economic models, which were calibrated against key variables in OECD economies. Thus, the economic dimension of our ABM integrates functions, processes and entities that were able to reproduce real macroeconomic trends (e.g. for debt dynamics) between 1970 and 2010 (see calibration in S3 Appendix). With regard to the environmental dimension, the ABM computes simple environmental equations, and processes, which are integrated into the economic system–thus forming the SES presented. Considering that there is no perfect model that works in all settings [52], our theoretical (environmental) algorithms and coefficients cannot be applied worldwide. Yet, the overall results obtained for the debt-sustainability relationship are considered to be reliable and stable to reasonable changes in specifications, above all in those countries with strong debt-sustainability relationships. Furthermore, our model provides a platform that can be further specified and applied across multiple empirical case-studies.

Answering our research question (see Introduction), the exploration of the viability and sustainability of the system modelled reveals its susceptibility towards instabilities related to monetary debt. Debt is an economic phenomenon that has been widely accepted by neoclassical and the so-called Keynesian approaches of the economy [53]. Our model shows that the current debt-driven monetary system creates the conditions in which continual economic growth–which is the overriding economic objective of most countries–becomes a necessity. The model of debt-fuelled growth requires ever-faster growth rates to allow the repayment of ever-increasing debt [54], and ever-faster growth requires, currently, an ever-increasing production and sale of goods and services, thus increasing the use of resources and emission of pollutants [7]. Under this context, natural resource sustainability is challenged by the depletion of natural resources driven by increasing debt stocks. Thus, the difficulty of avoiding collapse under these conditions may help explaining why monetary debt is a key factor with regard to (un)sustainable outcomes.

Interestingly, our results provide new insights to this debate. Our model shows that the economy does not grow or become unstable due to the debt burden enhanced by the monetary system, or the debt-based nature of the economic system itself–but rather this is the outcome of the *inappropriate use* that firms and speculators make of debt (i.e. bank credits). In fact, results show that non-debt-based systems (i.e. full-reserve) can also create unstable GDP trends over time, even in the absence of debt, thus showing that debt itself is not the main factor driving natural resource depletion. In particular, our fractional-reserve system scenario with early government intervention (i.e. 50%, Fig 2) shows that the use of debt, rather than the presence of debt in the system, is important to determine the grade of sustainability. Based on our results, we argue that the system does not impose a growth imperative *per se*, i.e. the debt-based economic system may not be, by definition, environmentally unsustainable. Rather, agents’ behaviour through the use of credits and the system’s dynamics show a tendency to increase natural resource unsustainability. In short, is not only the “*what*”–the (type of) system–that matters, but the “*how*”–the role of entities and credits in the system, and their relationships with the environment.

This idea aligns partially with the hypothesis proposed in this paper, which stated that highly-debt dependent economies exert negative effects on natural resource stocks. Yet, our results show that this is not due to debt itself, but the type of use that is made of it. The importance of this argument lies in the fact that one of the many criticisms of the monetary system is that the growth imperative is induced by the system itself because society receives less money (the principal of a loan) than that they have to pay back (principal + interest) [5]; this would induce agents to either monetize and liquidate the natural capital still available as unused resources, or to increase productivity. However, because the total factor productivity (TFP)–which refers to the portion of output not explained by the amount of inputs used in production–only grows at an average of 1.006% in the OECD including energy [55]–which could be increased to yield the required growth–, economic growth is achieved mainly by using greater stocks of natural resources. Thus, the profit-seeking behaviour of firms and speculative agents, i.e. see *Homo economicus* in [56], drives the inappropriate use of credits (debt), which consequently brings about systemic instability and negative implications for sustainable development.

In our model, the specific uses that firms make of credits are based on (i) processes related to asset speculation and (ii) exponential investments in technological development, both of them enhancing the disjunction between economic and environmental systems. The following sections analyse the implications of these elements for (de)coupling economic growth from environmental pressures.

### The speed of technological development

In the model, the government implements restrictive credit lending policies that prevent firms from further investing in technological development. Thus, technological efficiency–and with that, the production of goods–grows at a faster rate with no government intervention (see Fig 2). Unexpectedly, natural resource collapse, under no government intervention scenarios in fractional-reserve systems, occurs when technology efficiency shows lower values compared to full-reserve systems. It would have been expected that technological development should reach higher values in the former, due to higher investments through credits (see ‘Technology efficiency’, Fig 2). Therefore, collapses in the model are not specifically driven by the net peak values reached by technology efficiency (i.e. the higher the technological efficiency, the higher the chances for system collapses to occur), but rather by the *speed* (i.e. growth rate) at which technological development takes place. Thus, technology efficiency under full-reserve systems–with low investments in technological development–reaches a higher long-term net value compared to fractional reserve systems (with no government intervention), yet the speed of reaching this value is higher in the latter.

Moreover, the high rate of production efficiency under fractional-reserve systems (i.e. high debt stocks) encourages a mismatch between government’s capacity to implement conservation policies and the promotion of economic growth induced by firms. Thus, the slower pace at which conservation policies are implemented by the government is not sufficient to counterbalance the negative effects exerted on resources by faster technological development rates. In this regard, many OECD governments have been taking steps to adjust their policies to the growing technology and innovation [57], considering that technology efficiency and development have tended to accelerate over the last decades [58]. It should be highlighted that economists have usually resorted to technology and innovation as a source of ever-increasing efficiency and economic growth, regardless of the uncertainty and unpredictable nature of technological innovation [59]. However, technological progress is, in fact, a discontinuous process, where most significant innovations occur by “fits and starts” [59]. The discontinuous nature of technology has the risk of affecting the entire economic system and can lead to far-reaching changes in different social factors [60], as well as socioeconomic collapse [61]. Moreover, the Jevons Paradox establishes that increases in efficiency of resource use are usually outpaced by the rate at which consumption of those resources increases [62]. Overall, there is a tendency, in our society, to believe in technology despite the lack of support for this proposition; it is either an article of faith or based on statistically flawed extrapolations of historical trends [63].

Nevertheless, it is important to note that technological development can be applied to different fields and, therefore, have different implications for natural resource sustainability. In our model, technology refers to improving resource extraction efficiency and production processes, thus enhancing the above-noted negative environmental impacts. However, technological development focused on improving waste management (i.e. increase the amount of waste re-used and re-cycled), for instance, would probably be beneficial for the environment. Therefore, it is important to specify and analyse the particular use of technological development at the time of performing sustainability analysis. Under our particular context, a slower, yet constant, increase in technological development, focused on production efficiency (such as that shown under our full-reserve system scenario), could help creating win-win scenarios for GDP and natural resource availability.

### Speculation and price volatility

International policy makers and non-governmental actors have become increasingly concerned that the entry of speculators into the system might distort commodity prices by creating excess price volatility [64, 65]. In our model, the fractional-reserve banking scenario tends to create volatile, artificial and difficult-to-predict speculative markets. Thus, monetary debt is not used by the private sector to increase profits through increasing productivity and, thereby, benefit society (e.g. by enhancing technological efficiency); rather, it is mainly used by speculators to increase their own profits. Our results align with Keen [23], who states that money funding in the current debt-based economic system occurs according to speculation, instead of production-oriented goals–which enhances the possibility of economic collapses, instability and natural resource unsustainability, as shown by our model.

Scholars argue that the rapid increase of commodity derivatives and speculation globally is one financial actor affecting economic trends and, therefore, environmental sustainability [66]. The commodities for which derivatives are traded are numerous, including agricultural commodities (e.g. coffee, cocoa, soybeans, grains), crude oil and metals. Derivatives for these commodities are being traded in ever-increasing quantities globally due to the entry of new actors, such as large financial investors (e.g. pension funds, sovereign wealth funds) [65]. These have a limited interest in the underlying physical commodity, but instead invest in commodity derivatives as a means to diversify their investment portfolios and reduce investment risks [67]. Hyman Minsky’s Financial Instability Hypothesis, which has experienced a significant revival since the financial crisis of 2007–2009 [16], claims that, in prosperous times–when firms’ cash flow rises beyond what is needed to pay off debt–a speculative euphoria develops. Soon thereafter, debts exceed what firms can pay off from their incoming revenues, which in turn produces a financial crisis. A clear example of the impact of speculation on prices, debt and economic instability was observed in the U.S. oil market–see [68] and [69] for a detailed description.

Commodity price changes need to be linked to supply-demand processes and the availability of natural resources, rather than speculative processes. Our model shows the extent to which prices and demand processes, under fractional-reserve systems, are highly influenced by economic factors (i.e. the grade of speculation in the system), rather than environmental (i.e. resource availability). Thus, those periods when speculation follows positive increasing trends (see Fig 2) show weak coupling values between the economy (represented by the GDP) and the environment (represented by natural resource stocks). In contrast, those periods where artificial speculative markets are absent show contexts where economic elements are strongly coupled to the environment. Under low debt stocks, therefore, the market economy is highly influenced by the state of the environment, i.e. economic growth is aligned with the availability of natural resources, while the opposite is the case in systems with high debt-based speculative processes. It is important to reduce the level of speculation and speculative markets originated in the system, which could help moving towards decoupling GDP and natural resource use values.

### Government responses to natural resource unsustainability

Our results show that the economy does not necessarily have to grow or become unstable due to the debt burden encouraged by the monetary system; yet this is the common outcome because of the inappropriate use that firms make of credits, i.e. for speculative and the pace of increasing technology efficiency processes. In the model, this conflict is addressed by implementing government policies focused on enhancing natural resource conservation and more sustainable firm practices. In particular, late government intervention is not able to neither enhance a reduction of firms’ resource extraction rate nor increase resource replenishment rates. In this regard, a problem arises related to the difficulty to detect tipping points and predict environmental changes in complex coupled SES [70]. Complex systems are characterized for having multiple scales, non-linearity and interactive dynamics that are often unpredictable [71, 72]. Therefore, institutions have the difficult task of anticipating the complexity of SES dynamics over multiple temporal and spatial scales to avoid SES collapse, as seen, for example, in common pool resources, such as marine fisheries [73, 74]. In this regard, system unpredictability is enhanced not only by high technological development rates, but also due to speculation. As previously discussed, prices and demand processes are highly influenced by the grade of speculation in the system under fractional reserve systems, rather than by the availability of natural resources. Hence, high speculative debt-driven economies enhance the decoupling between economic and environmental systems.

The results obtained support the argument that the role of governments should be to invest in preventing market failures through environmental policies that focus on the long-term stability and resilience of the system. For instance, scholars argue that more resilient public institutions and governments are needed in order to be able to adapt to increasingly rapid technological advances [75]. Thus, a balance is likely needed, where the market still plays an important role in allocating resources efficiently, and the government balances this private perspective with an environmental one [76]. The problem here is that, under the current economic paradigm, seeking long-term objectives is penalized by a system focused on short-term gains, generally for the banking and private sector. Thus, increased opportunities should be given to the economic system to invest in both long-term environmental projects and short-term economic ventures, as compared to the situation in which money for loans is only created if it fulfils the profit criteria of private banks [77]. In real terms, it would reduce the ability of individual private actions to constantly expand the money supply and increase the economy’s debt burden, as well as halt environmental degradation reinforced by the private sector. Using climate change as an example, Nordhaus [78] argues that limited and gradual government interventions in the economy are necessary. Optimal regulation should reduce long-run growth by only a modest amount. Stern’s view [79] is less optimistic; it calls for more extensive and immediate interventions and argues that these interventions need to be in place permanently even though they may entail significant economic cost. The more pessimistic answers, such as those coming from degrowth economics [31, 80, 81], argue that, essentially, all growth needs to come to an end in order to save the planet. We argue that our results stand between Stern and Nordhaus viewpoints: gradual, yet not marginal, and strong interventions under business-as-usual scenarios are needed to prevent the economy from collapsing–not because the current debt-based market economy is, *per se*, unsustainable (as previously discussed), but rather because natural resource unsustainability is enhanced by agents’ and entities’ particular behaviours and dynamics.

## Conclusion

The results of our model show that debt-bearing economic systems can result in a complete collapse of both natural and economics systems. Debt is an enabling factor in the exploitation of natural resources for rational individual benefit and short-term gain, hindering long-term environmental and economic sustainability. However, our results show that debt-driven fractional-reserve economic systems do not impose a growth imperative *per se*, i.e. the debt-based system is not by definition unsustainable. Rather, the behaviour of entities and agents, and their decisions and relationships with regard to the environment, show a tendency to increase natural resource unsustainability. In the model, the particular uses that firms make of credits–causing the decoupling between GDP and resource availability–are based on (i) speculation, and (ii) exponential investments on technological development. Thus, it is argued that the profit-seeking behaviour of firms and speculative agents drives the inappropriate use of credits (debt), which consequently brings about systemic instabilities and negative implications for sustainable development.

The current version of the model should be considered as a conceptual tool that can be used to theoretically examine the relationship between debt and natural resource sustainability. Moreover, the model provides an analysis of the role of the monetary system in the economy and strongly suggests that macro-economic models should incorporate the banking sector if they are to become more relevant. Future versions of the model will include the integration of households as credit borrowers, thus including household speculation and desires. Furthermore, areas for improvement of the model include (1) disaggregating resources into ‘conventional’ (e.g. oil, food) and ‘non-conventional’ (e.g. timber), reflecting higher or lower household consumption dependences on such resources; (2) disaggregating conservation policies; and (3) introducing multiple coupled regions to represent countries with different policies.

## Supporting information

S1 Appendix Overview, Design Concepts and Details (ODD) Protocol. Standardized protocol describing the ABM in detail.
(PDF)

S2 Appendix Sensitivity analysis. Sensitivity analysis of the ABM, focused on analysing changes in model outputs with all parameters constant but the *critical-biomass-stock* parameter (for which a series of different values are considered).
(PDF)

S3 Appendix Model calibration. Calibration of the ABM, based on a comparative (qualitative) analysis between Keen’s (2009, 2010a) results and our ABM results.
(PDF)

## References

[1] Ripple WJ. World Scientists’ Warning to Humanity: A Second Notice. Bioscience. 2017; bix 125.

[2] Jackson T. Prosperity without Growth–economics for a finite planet. London: Routledge; 2009.

[3] Martinez-Alier J, Pascual U, Vivien F-D, Zaccai E. Sustainable de-growth: mapping the context, criticisms and future prospects of an emergent paradigm. Ecological Economics. 2010; 69: 1741–1747.

[4] Smith M, Hargroves KC., Desha, C. Cents and Sustainability—Securing Our Common Future by Decoupling Economic Growth from Environmental Pressures, London: Earthscan; 2010.

[5] Sorrell S. Energy, Growth and Sustainability: Five Propositions, Sussex Energy Group Conference ‘Energy transitions in an interdependent world’, 25–26 February. Sustainability. 2010; 2:1784–1809.

[6] Daly H. From a failed-growth economy to a steady-state economy. Solutions. 2010; 1:37–43.

[7] Huber J, Robertson J. Creating New Money–A Monetary Reform for the Information Age. 2000 London: New Economics Foundation.

[8] Korotayev AV, Tsirel SV. A Spectral Analysis of World GDP Dynamics: Kondratiev Waves, Kuznets Swings, Juglar and Kitchin Cycles in Global Economic Development, and the 2008–2009 Economic Crisis". Structure and Dynamics. 2010; 4(1): 3–57.

[9] Keen S. Solving the paradox of monetary profits. Economics: The Open-Access, Open Assessment E-Journal. 2010a; 4(2010–31): 1864–6042.

[10] Keen S. A model of endogenous credit creation and a credit crunch. Paul Woolley Financial Markets Dysfunctionality Conference, 2011.

[11] Lang DJ, Wiek A, Bergmann M, Stauffacher M, Martens P, Moll P, et al Transdisciplinary research in sustainability science: practice, principles, and challenges. Sustain Sci. 2012; 7: 25–43.

[12] Mauser W, Klepper G, Rice M, Schmalzbauer BS, Hackmann H, Leemans R, et al Transdisciplinary global change research: the co-creation of knowledge for sustainability. CurrOpin Environ Sustain. 2013; 5: 420–431.

[13] Fischer J, Gardner TA, Bennett EM, Balvanera P, Biggs R, Carpenter S, et al Advancing sustainability through mainstreaming a social-ecological systems perspective." Current Opinion in Environmental Sustainability. 2015; 14: 144–149.

[14] Redman CL, Grove JM, Kuby LH. Integrating Social Science Into the Long-Term Ecological Research (LTER) Network: Social Dimensions of Ecological Change and Ecological Dimensions of Social Change. Ecosystems. 2004; 7(2): 161–171.

[15] Berkes F, Folke C (Eds.). Linking Social and Ecological Systems: Management Practices and Social Mechanisms for Building Resilience. Cambridge University Press, New York; 1998.

[16] Keen S. Finance and economic breakdown: modeling Minsky’s financial instability hypothesis. Journal of Post Keynesian Economics. 1995; 17 (4): 607–635.

[17] Giraud G, Isaac FM, Bovari E, Zatsepina E. Coping With The Collapse: A Stock-Flow Consistent, Monetary Macro-dynamics of Global Warming. AIEE Energy Symposium; 2016.

[18] Ferber J. Multi-agent systems: an introduction to distributed artificial intelligence. Addison-Wesley Longman, Harlow, UK, 509; 1999.

[19] Balbi S; Giupponi C. Agent-Based Modelling of Socio-Ecosystems: A Methodology for the Analysis of Adaptation to Climate Change. International Journal of Agent Technologies and Systems. 2010; 2: 17–38.

[20] Filatova T, Verburg PH, Parker DC, Stannard CA. Spatial agent-based models for socio-ecological systems: challenges and prospects. Environ. Model. Softw. 2013; 45 (0): 1–7.

[21] Schulze J, Muller B, Groeneveld J. Grimm V. Agent-Based Modelling of Social-Ecological Systems: Achievement, Challenges, and a Way Forward. JASS. 2017; 20(2): 8.

[22] Antoniades A., Antonarakis A. and Schroeder P. (2017). Assessing the impact of Debt on Forest Cover, Air Pollution and Resource Efficiency. Sussex Sustainability Research Programme (SSRP), University of Sussex.

[23] Keen S. Household Debt‐the final stage in an artificially extended Ponzi Bubble, Australian Economic Review. 2009; 42: 347–357.

[24] Moss S. Agent-Based Modelling and Neoclassical Economics: A critical Perspective In Meyers R.A. (Ed.), Complex Systems in Finance and Econometrics (pp. 22–23). New York, NY: Springer; 2009.

[25] Farmer JD, Foley D. The economy needs agent-based modelling. Nature. 2009; 460(7256): 685–686. 10.1038/460685a 19661896

[26] Godley W, Lavoie M. Monetary Economics: An Integrated Approach to Credit, Money, Income, Production and Wealth. Basingstoke: Palgrave Macmillan, 2007.

[27] Tesfatsion L. Agent-based Computational Economics (ACE). 2006. Available from: http://www2.econ.iastate.edu/tesfatsi/ace.htm. Cited 14 Nov 2017.

[28] Hein E. Finance-dominated capitalism and re-distribution of income–a Kaleckian perspective. Cambridge Journal of Economics. 2014; 36: 325–354.

[29] OECD. Towards green growth–A summary for policy makers. OECD Meeting of the Council at Ministerial Level, 25–26 May 2011, Paris.

[30] Daly H. Towards an environmental macroeconomics. Land Econ. 1991; 67(2): 255–259.

[31] Jackson T, Victor PA. Does slow growth lead to rising inequality? Some theoretical reflections and numerical simulations. Ecological Economics. 2015; 121: 206–219.

[32] Politopoulos I. Review and Analysis of Agent-based Models in Biology. 2007. Available from: https://www2.csc.liv.ac.uk/research/techreports/tr2007/ulcs-07-021.pdf. Cited 13 Mar. 2018.

[33] North MJ, Macal CM. Managing Business Complexity: Discovering Strategic Solutions with Agent-Based Modelling and Simulation, Oxford: Oxford University Press; 2007.

[34] Abrahamson D, Blikstein P, Wilensky U. Classroom model, model classroom: Computer-supported methodology for investigating collaborative-learning pedagogy. Proceedings of the Computer Supported Collaborative Learning (CSCL) Conference. 2007; 8(1): 46–55.

[35] Brown DG; Robinson DT. Effects of heterogeneity in residential preferences on an agent-based model of urban sprawl. Ecology and Society. 2006; 11(1).

[36] Effken JA, Carley KM, Lee J.-S, Brewer BB. Verran JA. Simulating Nursing Unit Performance with OrgAhead: Strengths and Challenges. Computers Informatics Nursing. 2012; 30(11): 620–626. 10.1097/NXN.0b013e318261f1bb PMC3509226 22918133

[37] Epstein JM. Modelling civil violence: An agent-based computational approach. Proceedings of the National Academy of Sciences of the United States of America. 2002; 99(3): 7243–7250. 11997450 10.1073/pnas.092080199 PMC128592

[38] Gilbert N, Troitzsch KG. Simulation for the Social Scientist. Milton Keynes: Open University Press, Second Edition; 2005.

[39] Epstein JM. Generative Social Science: Studies in Agent-Based Computational Models. Princeton University Press 2006.

[40] Bonabeau E. Agent-based modelling: Methods and techniques for simulating human systems. PNAS. 2002; 99(3): 7280–7287. 12011407 10.1073/pnas.082080899 PMC128598

[41] DeAngelis DL and Mooij WM. Individual-based modelling of ecological and evolutionary processes. Annual Review of Ecology, Evolution, and Systematics. 2005; 36: 147–168.

[42] Grimm V. Ten years of individual-based modelling in ecology: what have we learned and what could we learn in the future? Ecological Modelling. 1999; 115(2–3): 129–148.

[43] Graziani A. The Theory of the Monetary Circuit. Economies et Societes. 1990; 24(6): 7–36.

[44] Bruun C; Heyn-Johnsen C. The Paradox of Monetary Profits: An Obstacle to Understanding Financial and Economic Crisis? Managing Financial Instability in Capitalist Economies. 2009; 25.

[45] Wilensky U. NetLogo [computer software]. Center for Connected Learning and Computer-Based Modeling. Northwestern University, Evanston, IL 1999 Available from: http://ccl.northwestern.edu/netlogo. Cited 13 October 2017.

[46] Grimm V, Berger U, Bastiansen F, Eliassen S, Ginot V, Giske J, et al A standard protocol for describing individual-based and agent-based models. Ecological Modelling. 2006; 198(1–2): 115–126.

[47] Grimm V, Berger U, DeAngelis DL, Polhill JG, Giske J, Railsback SF. The ODD protocol: a review and first update. Ecological Modelling. 2010; 221(23): 2760–2768.

[48] Global Footprint Network. Glossary–Biological capacity or biocapacity. 2018. Available from: footprintnetwork.org/resource/glossary. Cited 11 Apr 2018.

[49] European Central Bank. The Monetary Policy of the ECB. 2011 [online]. Available from: https://www.ecb.europa.eu/pub/pdf/other/monetarypolicy2011en.pdf. Cited 16 Apr 2018.

[50] ten Broeke G, van Voorn G. Ligtenberg A. Which Sensitivity Analysis Method Should I Use for My Agent-Based Model? Journal of Artificial Societies and Social Simulation. 2016; 199(1): 5.

[51] Ritter FE, Schoelles MJ, Quigley KS, Klein LC. Determining the number of simulation runs: Treating simulations as theories by not sampling their behaviour In: Narayanan S. and Rothrock L. (Eds.). Human-in-the-loop Simulations: Methods and Practice. London: Springer-Verlag: pp. 97–116; 2011.

[52] Ostrom E. A diagnostic approach for going beyond panaceas. Proceedings of the National Academy of Sciences of the United States of America. 2007; 104: 15181–15187 10.1073/pnas.0702288104 17881578 PMC2000497

[53] Keen S. Straight Talk with Steve Keen: It’s All About the Debt (by Adam Taggart). 2010b. Available from: https://www.peakprosperity.com/blog/straight-talk-steve-keen/47466#_ftn1. Cited 18 Nov 2017.

[54] Daly H. Growth, Debt and the World Bank. Ecological Economics. 2011; 72: 5–8.

[55] Murillo-Zamorano LR. Total Factor Productivity Growth, Technical Efficiency Change and Energy Input. An International Frontier Analysis. The University of York Discussion Papers in Economics No. 2003/09; 2003.

[56] Persky J. Retrospectives: The Ethology of Homo Economicus. The Journal of Economic Perspectives. 1995; 9(2): 221–231.

[57] OECD. Science, Technology and Innovation in the New Economy–Poly Brief. 2000. Available from: http://www.oecd.org/science/sci-tech/1918259.pdf. Cited 23 Jan 2018.

[58] Modis T. Forecasting the growth of complexity and change. Technological Forecasting and Social Change. 2002; 69(4):377–404.

[59] Lafforgue G. Stochastic technical change, non-renewable resource and optimal sustainable growth. Resource and Energy Economics. 2008; 30(4): 540–554.

[60] Helpman E, ed. General Purpose Technologies and Economic Growth. MIT Press, Cambridge MA; 1998.

[61] Diamond J. Collapse: How Societies Choose to Fail or Succeed. Viking press, United States; 2005.

[62] Jevons WS. The Question of Coal. Macmillan, London; 1985.

[63] Brown JH, Burnside WR, Davidson AD, DeLong JP, Dunn WC, Hamilton MJ, et al Energetic Limits to Economic Growth. BioScience. 2011; 61(1): 19–26.

[64] Cox CC. Futures trading and market information. J. Polit. Econ. 1976; 84: 1215–1237 24.

[65] UNCTAD. Price Formation in Financialized Commodity Markets–The Role of Information, United Nations; 2011.

[66] Galaz V, Pierre J. Superconnected, Complex and Ultrafast: Governance of Hypoerfunctionality in Financial Markets. Complexity, Governance & Networks. 2017; 3(2): 12–28.

[67] Gorton G, Rouwenhorst KG. Facts and fantasies about commodity futures. Financ. Anal. J. 2006; 62: 47–68.

[68] Clark J. How does oil speculation raise gas prices? HowStuffWorks–Stock. 2016. Available from: https://money.howstuffworks.com/stock-market-channel.htm. Cited 29 Jan 2018.

[69] PCI–Permanent Subcommittee on investigations. The Role of Market Speculation in Rising Oil and Gas prices: A Need to Put the Cop Back on the Beat. 2006. Available from: https://www.hsgac.senate.gov/imo/media/doc/SenatePrint10965MarketSpecReportFINAL.pdf. Cited 16 Apr 2018.

[70] Dawson TP, Rounsevell MDA, Kluvánková-Oravská T, Chobotova V, Stirling A. Dynamic properties of complex adaptive ecosystems: implications for the sustainability of service provision. Biodiversity and Conservation. 2010; 19(10): 2843–2853.

[71] Axelrod R, Michael DC. Harnessing Complexity: Organizational Implications of a Scientific Frontier. Reprint edition. New York: Basic Books; 2001.

[72] Holling CS, Berkes C, Folke C. Science, Sustainability, and Resource Management In: Berkes F. and Folke C. (Eds.) Linking Social and Ecological Systems: Management Practices and Social Mechanisms for Building Resilience, pp. 342–362. Cambridge, UK: Cambridge University Press; 1998.

[73] Beddington JR, Agnew DJ, Clark CW. Current problems in the management of marine fisheries. Science. 2007; 316:1713–1716. 10.1126/science.1137362 17588923

[74] Hardin G. The tragedy of the commons. Science. 1968; 162: 1243–1248. 5699198

[75] Lebel L. Governance and the Capacity to Mange Resilience in Regional Social-Ecological Systems. Ecology and Society. 2006; 1191: 19.

[76] Stiglitz DJ. Moving beyond market fundamentalism to a more balanced economy. Annals of Public and Cooperative Economics. 2009; 80(3): 345–360.

[77] Koslowski P. The Ethics of Banking On the Ethical Economy of the Credit and Capital Market, of Speculation and Insider Trading in the German Experience In: The Ethical Dimension of Financial Institutions and Markets. Springer, Berlin, Heidelberg; 1995.

[78] Nordhaus WD. A review of the Stern Review on the economics of climate change. Journal of Economic Literature. 2007; 45: 686–702.

[79] Stern N. The Economics of Climate Change: The Stern Review Cambridge and New York: Cambridge University Press; 2007.

[80] Meadows DH, Meadows DL, Randers J. Limits to Growth: The 30-Year Update. Chelsea Green Publishing Co., White River Junction, Vermont; 2004.

[81] Victor PA, Rosenbluth G. Managing without growth. Ecological Economics. 2007; 61: 492–504.

