# Correction: It's not the '*what*', but the '*how*': Exploring the role of debt in natural resource (un)sustainability

**DOI:** 10.1371/journal.pone.0202509

**Published:** 2018-08-13

**Authors:** Julen Gonzalez-Redin, J. Gareth Polhill, Terence P. Dawson, Rosemary Hill, Iain J. Gordon

Fig 2 is incorrect. The authors have provided a corrected version here.

**Fig 2:**
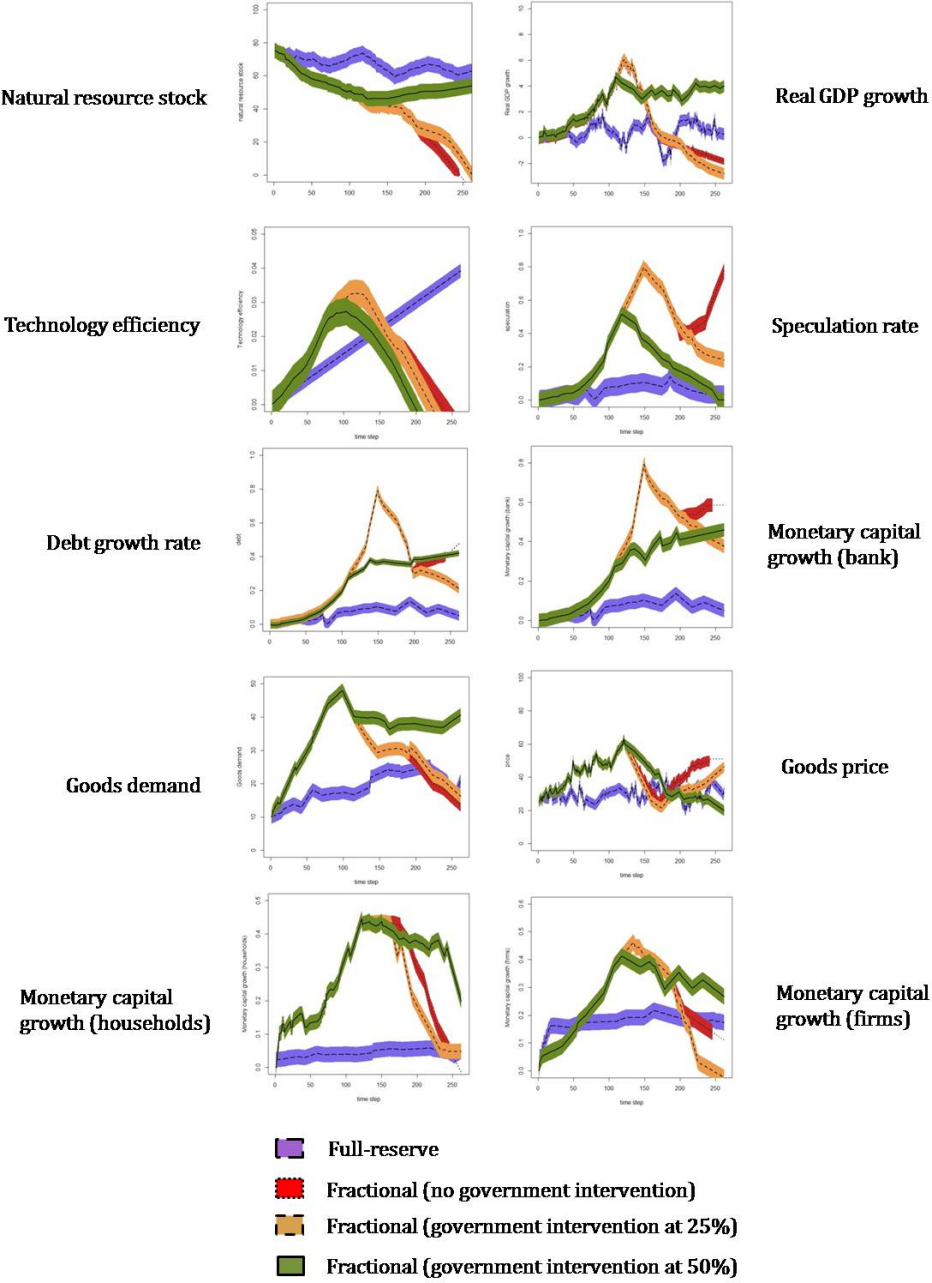
Simulation results. Results obtained for the indicators selected under a fractional-reserve system–without government intervention (red dotted line) and with government intervention when the total natural resource stock is at 25% (yellow short-dash) and 50% (green solid line)–and under a full-reserve system (purple long-dash line). Black coloured curves (i.e. dotted, solid, short and long-dashed) show the mean values, whereas coloured bands represent the standard error bars including all the runs computed for each indicator under every scenario.
