# Supplementary material for: It's not the 'what', but the 'how': Exploring the role of debt in natural resource (un)sustainability
Source: PLoS One. 2018 Jul 20;13(7):e0201141. doi: 10.1371/journal.pone.0201141 (PMC6054380; doi:10.1371/journal.pone.0201141)
Supplement: S1 Appendix — Standardized protocol describing the ABM in detail. (PDF) [file pone.0201141.s001.pdf]

S1 Appendix:

Overview, Design Concepts and Details (ODD) Protocol

1.1 Purpose

Described in main text.

1.2 Entities, state variables, and scales

Described in main text.

Fig A shows the UML class diagram, while Table A shows the description of the state variables of the entities.

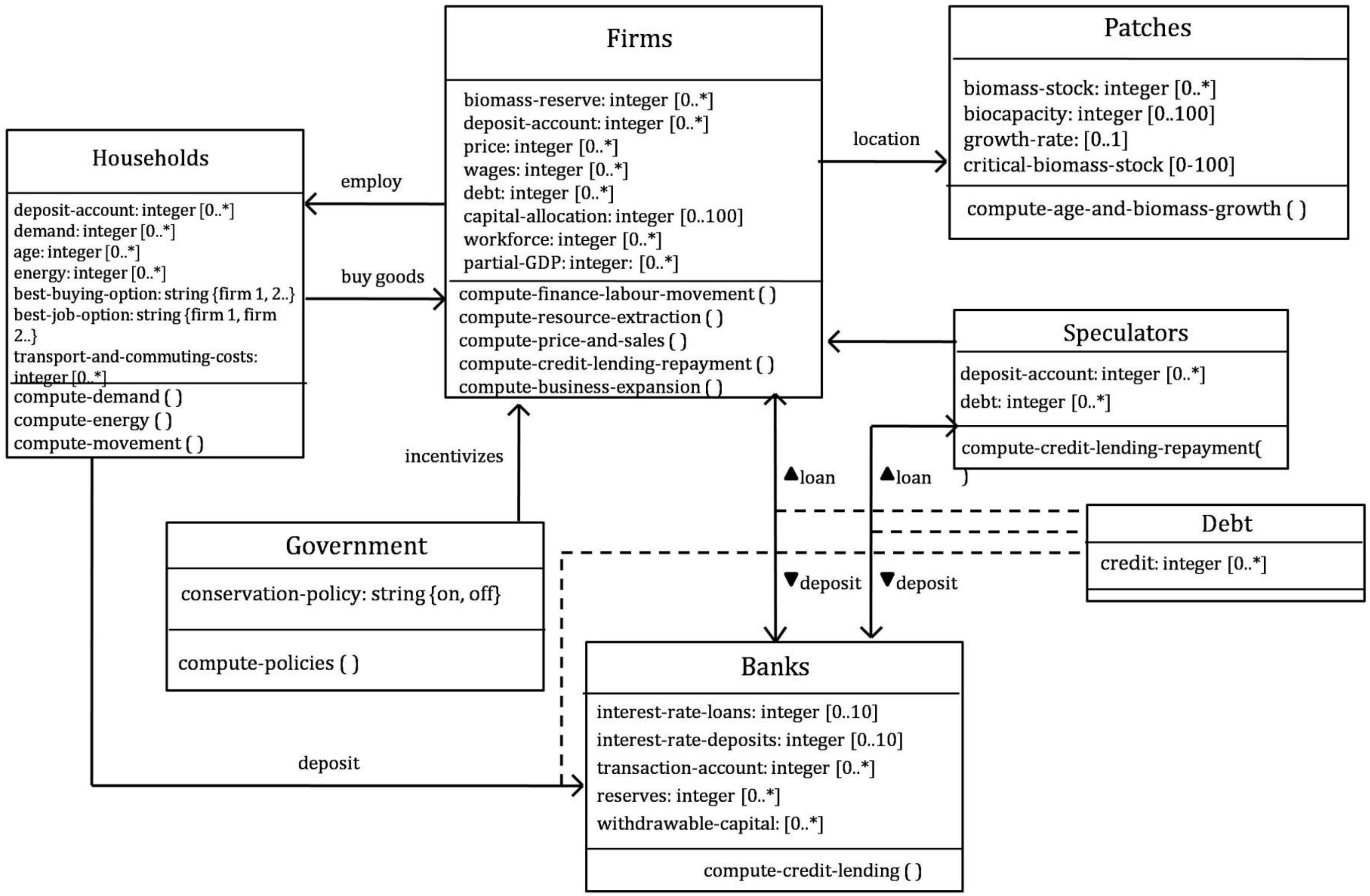

Fig A. UML Class Diagram. Structure diagram showing the system’s classes, their attributes, attribute values, functions/operations and relationship between classes. *Source*: author

Table A. Entities included in the model, their state variables, and whether these are exogenous (i.e. exogenously set by the observer/user, or endogenously generated by the model) and/or constant (i.e. values do not change during the simulation) variables.

State variable	Description	Exogenous?	Constant?	Initial value
	environment			
biomass-stock	Number of resource units available in patch.	No	No	0 – max-biomass-stock
biocapacity	Capacity of ecosystems to produce useful biomass and to absorb waste biomass generated by firms during extraction processes.	No	No	See 'Submodels'
crit-biomass-stock	Minimum threshold of biomass below which resources are unable to re-grow	Yes	Yes	10^3
max-biomass-stock	Maximum biomass value in each patch	Yes	Yes	10^9
growth-rate	Intrinsic growth rate of biomass	Yes	Yes	0 – 1
equivalence-factor	Productivity-based scaling factor that converts a specific land type (e.g. forest) into a universal unit of biologically productive area, a global hectare.	Yes	Yes	1.28
yield-factor	Factor that accounts for differences between countries in productivity of a given land type (e.g. cropland)	Yes	Yes	1.82
	government			

State variable	Description	Exogenous?	Constant?	Initial value
compute-government-policy	Activate government conservation responses	Yes	Yes	4 options/ scenarios
	firms			
deposit-account-firm	Monetary capital of firms	No	No	10^6 - 10^{30}
capital-technological-progress (CTP)	Amount of monetary capital allocated to technology-account by each firm, which is invested in new technologies to increase resource extraction efficiency.	No	No	0.15
capital-wages (CW)	Amount of monetary capital allocated to wages-account by each firm, which is invested in employee wages.	No	No	0.3
capital-equipment-materials (CEM)	Amount of monetary capital allocated to equipment-material-account by each firm, which reflects expenses for fuel, material and equipment	No	No	0.2
sales-niche	Chooser showing 4 integers which determines the radius size to be considered by firms when counting the number of households in such radius at the time of moving to another patch due to lack of employees	Yes	Yes	maximum
biomass-conversion-	Conversion rate of biomass (resources) into goods	Yes	Yes	0.5

State variable	Description	Exogenous?	Constant?	Initial value
ratio				
biomass-reserves	Physical deposit of each firm, where harvested resources are stored. Enables firms to cope with periods of an excess demand.	No	No	0
credits/debt	Loans borrowed by firms from the bank	No	No	0
credits-repaid	Amount of repaid loans by firms so far	No	No	0
credits-remaining	Amount of unpaid loans by firms so far	No	No	0
workforce	Total number of employees in each firm	No	No	nº households / nº firms
nominal-wage (NW)	Wages paid by firms to households	No	No	$10^3 - 10^5$
accelerator-effect	Adaptation of firms to household demand by increasing/decreasing resource extraction	Yes	Yes	3
price (P)	Firms' sales price	No	No	50 – 200
	households			
deposit-account-household	Bank deposit account of households.	No	No	$10^4 - 10^5$

State variable	Description	Exogenous?	Constant?	Initial value
max-age	Current age of each household.	No	No	90
energy	Energy obtained per resource unit consumed by households.	No	No	0 – 100
transport-cost	Cost of mobility for buying goods.	Yes	No	10
commuting-cost	Cost of mobility for commuting to work	Yes	No	10
demand	Each household's demand over goods	No	No	0
aggregate-goods-demand (AGD)	Variable that computes the total individual-goods-demand on firms by households	No	No	0
		bank		
withdrawable-capital	Capital available for withdrawable by households for good consumption	No	No	$\sum$.household deposit accounts
cash-reserve-ratio (CRR)	Proportion of money allocated as withdrawable-capital	Yes	Yes	0.02 – 1
bank-reserve (vault)	Bank's money reserve, where repaid loans are saved and from which loans are lent out	No	No	household deposit accounts * (1 – CRR)
credit-lending-	Frequency at which credits are lent our by the bank to firms	No	Yes	12 (months)

**State variable**	**Description**	**Exogenous?**	**Constant?**	**Initial value**
frequency				
credit-repay-frequency	Frequency at which credits are paid back from firms to the bank	No	Yes	15 (months)
interest-loans	Interests on loans that firms and households pay to the bank	Yes	Yes	0.05
interest-deposits	Interests on deposits that the bank pays to households	Yes	Yes	0.02
	speculators			
credits-borrowed	Amount of credits borrowed from the bank	No	No	0
speculation-debt	Debt owed by speculators to the bank	No	No	0

1.3 Process overview and scheduling

Described in main text.

1.4 Design Concepts

1.4.1. Basic Principles

Steve Keen's (2009, 2010a) pure credit economic models are used as a basis to simulate the economic dimension of our ABM. Keen's models uses the Monetary Circuit Theory as a framework, an heterodox theory of monetary economics often associated with the post-Keynesian school that permits to examine the role of banks and debt through a simple monetary platform – elements that are usually ignored by mainstream economists (Keen, 2009).

1.4.2. Collectives

The bank agent represents a group of commercial banks lending credits to firms, which use them to fund extraction of natural resources. The government represents those policies, implemented at different governmental scales, focused on enhancing environmental conservation of natural resources.

1.4.3. Emergence

Model outcomes are grouped into environmental and socio-economic. Environmental outputs consist of biomass stock (i.e. natural resource stock) and biocapacity, i.e. patch variables. The socio-economic outputs consist of GDP (observer-variable), technology efficiency (firm variable), debt growth rate (firm-variable); price (firm-variable); demand (household-variable) monetary capital stock (firms, household and bank variables); goods price (firm-variable), goods demand (household-variable) and speculation rate (speculator-variable).

1.4.4. Adaptation

Adaptation is to some degree reflected in the different rules for movement by household and firm agents. Firms move to the closest patch with highest biomass stock and higher number of households in neighbour patches when labour is zero, or when their biomass stocks drop under an exogenously set threshold. Households' movement is based on prioritizing firms nearby with low prices and high wages. Both firms and households adapt to negative financial situations by borrowing bank credits. Similarly, speculator agents adapt to prices in order to borrow more or less (or none) bank credits. Finally, the government agent adapts to the environmental status of natural resources overall for implementing conservation policies.

1.4.5. Objectives

The objectives are implicit to agents. Agents' decision-making algorithms enhance profit-seeking behaviours aimed at increasing monetary capital.

1.4.6. Learning

Agents change several adaptive traits during simulation, making decisions based on the information obtained from different model parameters. For instance, *credit-lending-frequency* and *credit-repay-frequency* are parameters that change their value based on the type of economic scenario modelled. Thus, the rates at which firms repay their loans vary from scenario to scenario, as well as the capital available for credit lending. Another example is given by the *nominal-wages* parameter, which influences household consumption in the goods market.

1.4.7. Prediction

Firms predict a potential future depletion of resources when these drop below a critical threshold, thus adapting their resource extraction efforts in concordance. Similarly, the government considers potential future scenarios of environmental and/or economic collapse and implements conservation policies in consequence.

1.4.8. *Interaction*

The bank directly interacts with firms and households via the credit market. Households directly interact with firms within the labour and goods markets. Firms directly interact between them via competition for resources and credits. Households interact between them via competition for low good prices and high wages.

1.4.9. *Observation*

Graphical output on the NetLogo interface shows the stock of resources of each patch, via patch colour. Firms (grey agents with 'factory' shape) and households (red agents with 'person' shape) are also displayed. Each time step, the model displays a trace on the movements of all agents so that their movements can be observed.

To allow the observation of the generated environmental and socio-economic effects, summary statistics are provided via reporters and plots in the interface and output files. The model provides graphical display of several model outputs.

1.5 Initialization

The initial landscape consists of a grid of 100 × 100 patches. The location of the bank, government and speculators is randomly set, yet irrelevant for model outcomes. Each household and firm agent is assigned a random (patch) location, where one firm and no more than three households can coincide in each patch. See Table A for the initial values for all the parameters computed.

1.6 Input Data

The interest rate on credits and deposits follows historical data (1967-2000) for the UK (World Bank, 2012). The Yield Factor (YF) refers to the difference in production of a given land type across different nations, measured in kg/ha, while the Equivalent Factor (EQF) translates a specific land type (e.g. cropland, pasture, forest, fishing ground) into a universal unit of biologically productive area (ha).

1.7 Submodels

Most algorithms are adapted from Keen (2010) to our particular modelling context by disaggregating his equations and algorithms (computed by homogenous entities) for each heterogeneous agent in our model.

1.7.1 Scenario selection

The observer/user selects the scenario to be computed. The Result section in the main paper describes the scenarios modelled, based on economic systems with different c*ash-reserve-ratios,* going from the lowest *fractional reserve banking* (2%) to a *full reserve banking* (100%) system. The lower the cash reserve ratio, the higher the fraction of the commercial bank's monetary capital available for credit lending to firms. Through this approach, we want to study the environmental impacts of systems with different debt dynamics and debt stocks available for borrowing.

1.7.2 Patches compute biomass-stock

Each land parcel (patch) computes one *resource stock (Rs)*, which increases based on a *growth rate* parameter, and decreases due to the *resource-extraction* process of firms. Growth-rate values are halved when resource stocks drop below a *critical resource threshold*, since the capacity of resources to re-grow is affected.

The biological demand on each patch is determined by its *biocapacity*:

$$B = Rs \cdot YF \cdot EQF \tag{1}$$

where YF is the *yield factor* and EQF the *equivalent factor* (same for all patches, see 'Input Data' above). While most applied YF and EQF functions use hectares as a unit, we use the abundance of resources in each patch as our own particular unit; this is done in order to enhance B variability between patches, since all patches in our model have the same number of hectares.

1.7.3 *Firms extract resources*

Firms impact on each patch through resource extraction. First, firms calculate the amount of resources to be extracted in the following time step based on the quantity of goods needed to meet the demand (note that firms use the demand data from the previous time step), labour (i.e. workforce) and the surplus resources available in their own current biomass reserve, obtained from previous extractions; BR permits each firm to cope with periods of excess of demand, due to temporal lags between resource extraction and good consumption by households, or when resources are depleted, thus allowing firms to sell goods available from their BR. Finally, firms calculate the amount of resources to be extracted (Re):

$$R_e = (D \cdot L \cdot c) - B_r \tag{2}$$

where D refers to households' demand, L to *labour*, c to a *biomass conversion factor,* and Br to the *biomass reserve* (in tons). Firms decrease their extraction efficiency (and, therefore, the amount of resources extracted) when the biomass stock in their current patch is lower than a critical resource threshold. This represents the increasing technological and capital difficulties of extracting resources with low stocks left, e.g. extracting unconventional oil requires a higher investment of capital and more developed technologies.

1.7.4 *Households compute good consumption, movement and energy input/outputs*

Households consume goods from the closest firm with lowest *price* (see 'Firms compute price' below). The number of goods consumed at time t depends on each household's *demand (D):*

$$D = \frac{H_c}{(P \cdot v)} \tag{3}$$

where Hc refers to households' total *monetary capital*, P refers to each firm's price and *v* is the *accelerator effect*. The accelerator effect is related to the GDP, where an increase of the latter enhances firms' capital investment. In the real-world, when there is an excess of demand, firms typically have two options: (i) to decrease demand by

raising prices, or (ii) to increase investment in capital goods to the level of demand. Due to the accelerator theory stating that firms typically choose to increase production – which enlarges capital stock and increases profits – our model only considers an increase of investments in good production by firms in order to meet demand. Therefore, the accelerator effect represents the adaption of firms to households' goods demand through increases/decreases in investments for good production. Thus, the link between household demand and firms' capital investments is what creates the accelerator effect and, together, drive a continued and exponential economic growth in our model. An example is given by the current increasing demand for capital goods (e.g. wind turbines) which is driven by the demand for consumer goods (e.g. renewable energies), this giving rise to the accelerator effect.

In our model, firms try to predict the investment required (*K*) each time step in order to generate enough goods (and have them in stock) that are able to meet household demand (*D*). Thus:

$$K = (AGD_{t-1} \cdot L \cdot F_c \cdot M_c) \tag{4}$$

where *AGD* is the *aggregate demand of goods* by households regarding firm f, L is *labour* of firm f (as the number of employees working for firm f), *Fc* is the *monetary capital* of firm f, and *Mc* refers to extraction-demand correction mechanism. AGD considers the AGD trend over the past time steps. Thus, if the average AGD over the last time steps has been higher than the total average AGD since beginning of simulation, and if at least half of the highest AGD values have been computed during the last time steps, firms expect a higher household demand (D) for the following time step compared to the previous. MC is a mechanism computed by firms in order to improve on past failures regarding the prediction of future D; thus, if the D prediction of firm f the previous time step was not precise enough, i.e. more resources than finally demanded were actually extracted by firm f, this correction mechanism enables firm f to invest less capital in the following time step.

Consumed goods provide households with energy based on an *energy converter* function, which states the energy units gained by households based on the amount of goods consumed. The accumulated energy is consumed by households every time step

through their *metabolism*. When a minimum amount of energy (exogenously set by the parameter *birth required energy)*, age (set by *minimum birth threshold*), and monetary capital (set by *capital-threshold*) is reached, household reproduce. Households die if they run out of energy or reach a *maximum age*.

Households perform a combined movement based on *commuting distance weight* and *consumption distance weight*. Thus, each household moves to the closest patch where the firm with the highest nominal wage and lowest price is located. *Commuting* and a *shopping transport costs* are charged to households.

1.7.5 Firms compute price

All firms sell the same type of good, yet each firm sets a specific *price*. The price (P) links the monetary flow and the physical output produced by firms, thus:

$$P = \frac{D \cdot P_k}{B_r} \tag{5}$$

being D the demand on each firm by households, B_r the biomass reserve, and P_k a speculation rate (see below). Households pay the corresponding price to firms, which is subtracted from their deposit accounts and transferred to firms' accounts. The firm offering the lowest price is placed at the top of a right-skewed distribution (showing price on the X Axis and demand in the Y axis), thereby being the one prioritized by households for consumption. Each firm has a maximum number of households to which it can sell goods to (i.e. every time step), thus preventing households to buy goods from the same firm (i.e. the one with the lowest price. Note that a *minimum price* and *maximum price* is exogenously set for all firms in order to ensure a control over the prices that households pay for consumption. In addition to this, households also prioritize those firms closer to their current location.

1.7.6 Firms compute labour and finance

Each firm computes *capital-management* in order to invest the monetary capital from its *deposit-account* in three different aspects, namely *capital-wages (CW), capital-*

technological-progress (CTP) and *capital-equipment-materials (CEM).* The proportion of the deposit-account allocated for each of them is always less than 1. The remaining capital in the deposit account is kept by firms for potential business expansion processes (see 'Business expansion').

Firms compute *productivity (p)* as the effectiveness of productive effort measured in terms of the rate of output per unit of input:

$$p = \frac{F_{c_t} - F_{c_{t-1}}}{L} \quad (6)$$

where $F_{c_t} - F_{c_{t-1}}$ are the profits obtained from one year to the next one, and L refers to labour, i.e. the number of employees working for each firm. Productivity is computed by firms to calculate employees' nominal wages (see below).

Each firm sets a *nominal wage* (W_n), given by the function:

$$W_n = \frac{W_c \cdot F_c}{L} \quad (7)$$

where W_C refers to capital wage, i.e. the per cent value of total capital allocated for wages, F_c to the firm's deposit account (i.e. monetary capital), and L to labour. For the sake of simplicity, it is assumed that all the employees working for the same firm earn the same wage, due to our interesting lying in the amount of capital allocated for wages instead of individual wages or wealth distribution. Nominal wages vary within a range established by an exogenously set *minimum* and *maximum wage*. Households work for the closest firm offering the highest wage. Each household receives a nominal wage in a time-step-basis in its deposit account.

Each household considers working for another firm every month, thus households can either remain in the same firm during the entire simulation or change every several time steps. Firms with no employees perform a market analysis, in order to seek ways to attract more employees; this is represented in our model by firms moving to empty patches with high number of households in their surrounding patches. A *transport cost* is subtracted from each firm's deposit account during this process. Those firms with

zero labour that cannot find any empty patch (thus remaining in the same location) disappear from the simulation. Similarly, firms move to a new resource extraction point (patch) if the sum of the resource stock in their current land parcel and their biomass reserves is lower than those goods needed to meet household demand for one entire year. The new location is the empty patch (i.e. with no other firms) with highest stock of resources, for which a *transport-cost* is charged. If biomass reserves and resource stocks are depleted firms are go bankrupt.

1.7.7 Banks compute finance

The bank possesses two stocks of monetary capital: *withdrawable capital (WC)* and *bank reserves (BR).* The prior contains the amount of household deposits times the cash reserve ratio (from 2 to 100, depending on the banking system selected). The bank reserves contains the amount of money remaining, which in a 2% fractional reserve banking system would be 98% of household deposits, plus those credits repaid by firms to the commercial bank. In a full reserve banking system bank reserves only contains the credits repaid.

The bank pays deposit interests to households in a time-step-basis. The money is transferred from the bank's reserves to households deposit accounts based on an *interest rate*. Thus, the commercial banks make profits due to the surplus generated from the difference between the total amount of interests on deposits paid to households and the total amount of interests on credits received from both firms and households.

1.7.8 Firms borrow credits

Under a credit-based-economy, each firm considers borrowing one credit from the bank if the mean deposit account from the previous time steps is higher than the current deposit account value. The amount borrowed is based on the capital needed to cover the expenses for one entire year (48 time steps), that is, the sum of *CTP*, *CEM* and *CW*. The credit is loaned in a time-step-basis for one entire year.

1.7.9 Firms consider business expansion

When each firm's deposit account is higher than a *relative-wealth* threshold, firms expand their business by creating one new firm in the closest patch with highest biomass stock – the mother firm is wealthy enough to select and secure a favourable niche for its new branch to start up. A *transport-cost-new-extraction-point* is charged to the new firm. Business expansion by firms is not directly funded using monetary capital from their deposit accounts, but rather by borrowing one credit from the commercial bank. This credit is used to cover the costs of transport and expanding their business, thus firms can keep investing in extracting more resources. Borrowed credits are repaid as above-explained.

1.7.10 Speculators compute speculation

Speculators purchase derivatives, i.e. instruments to bet on what price the asset will reach by a future date. Speculators have no hand in the sale of the commodity they are betting on, i.e. they are not the buyer (households) or the seller (firms). The rate of Ponzi speculation is a non-linear function of the rate of growth.

$$P_k = k_g \cdot Y \tag{9}$$

where k_g is the Ponzi behaviour (i.e. economic growth rate) and Y the *model output* (i.e. goods produced per time step). Thus, price variability and speculation are related elements (see 'Firms compute price' above), as well as speculation and GDP (i.e. growth rate). Based on Keen (2009), asset price inflation in euphoric economy phases creates a suitable context to make profits by trading assets on a rising market. In particular, this gives rise to a class of speculators that Minsky called 'Ponzi financiers'. Thus, when price inflation reaches an exogenously set minimum threshold, the model 'generates' speculator agents that start trading on financial assets. These speculators are willing to incur debts (i.e. speculators borrow credits from the bank). In particular, since they expect to be able to sell on the assets at a profit, speculators incur debts that exceed their capital at the time of borrowing credits. If credit interest rates overcome an exogenously set maximum threshold (note that the model includes an input function

interests at a certain rate), speculators start selling the purchased assets in order to be able to repay their debts (see below). These assets are sold to those speculators with high monetary capital that are not yet interested in selling their assets, but willing to keep purchasing more. Therefore, simulations may include speculators with different financial situations. As a result, additional sellers enter the asset market, thus raising the prices in which the Ponzi financers depend.

The sensitivity analysis performed showed that the values 25 and 50 of the critical-biomass-stock parameter are critical thresholds regarding model outputs for natural resources and real GDP growth indicators.

1.7.11 Speculators compute speculation

Speculators and those firms in debt with the bank start repaying the credit (with interest, following an exogenously set *interest rate*) when their current deposit account is equal or higher than the mean deposit account from the previous year. The full amount of credit, plus interests, are repaid in a time-step-basis to the bank's reserves. Under a credit-based economy, if firms and speculators are under an economic downturn but bank reserves are empty, firms go bankrupt and are removed from the simulation.

1.7.12 Government computes conservation policies

The government agent computes various policies in order to halt resource depletion and avoid economic collapses (described in main text).
