# Supplementary material for: It's not the 'what', but the 'how': Exploring the role of debt in natural resource (un)sustainability
Source: PLoS One. 2018 Jul 20;13(7):e0201141. doi: 10.1371/journal.pone.0201141 (PMC6054380; doi:10.1371/journal.pone.0201141)
Supplement: S2 Appendix — Sensitivity analysis of the ABM, focused on analysing changes in model outputs with all parameters constant but the critical-biomass-stock parameter (for which a series of different values are considered). (PDF) [file pone.0201141.s002.pdf]

Sensitivity Analysis

A sensitivity analysis was performed on the *critical-biomass-stoc*k parameter to test the sensitivity (to this parameter) of 'Real GDP growth' and 'Natural resource stock' indicators. A set of predefined values were selected for the *critical-biomass-stock* parameter, and tested against the above-noted two indicators. These consisted of 12 values almost uniformly sampled in the range [0, 100], i.e. {15, 25, 35, 45, 47.5, 50, 52.5, 55, 65, 75, 85, 95}. Each *critical-biomass-stock* value (i.e. 12 in total) was run 100 times – making a total of 1200 runs – which is considered a reasonable number of runs to generate valid and stable predictions in stochastic simulations (Ritter et al., 2011).

As an example, Fig S2 shows a graphical representation of the sensitivity results obtained for the first six different *critical-biomass-stock* values from the list (i.e. 15, 25, 35, 45, 47.5, 50), with regards to the 'Natural resource stock' parameter. Note that the sensitivity results obtained for the values comprised between 50 and 100 (i.e. 52.5, 55, 65, 75, 85, 95) are not included in Fig S2; this is because all of them showed positive increasing trends – similar, yet higher, to those regarding *critical-biomass-stock* 50.

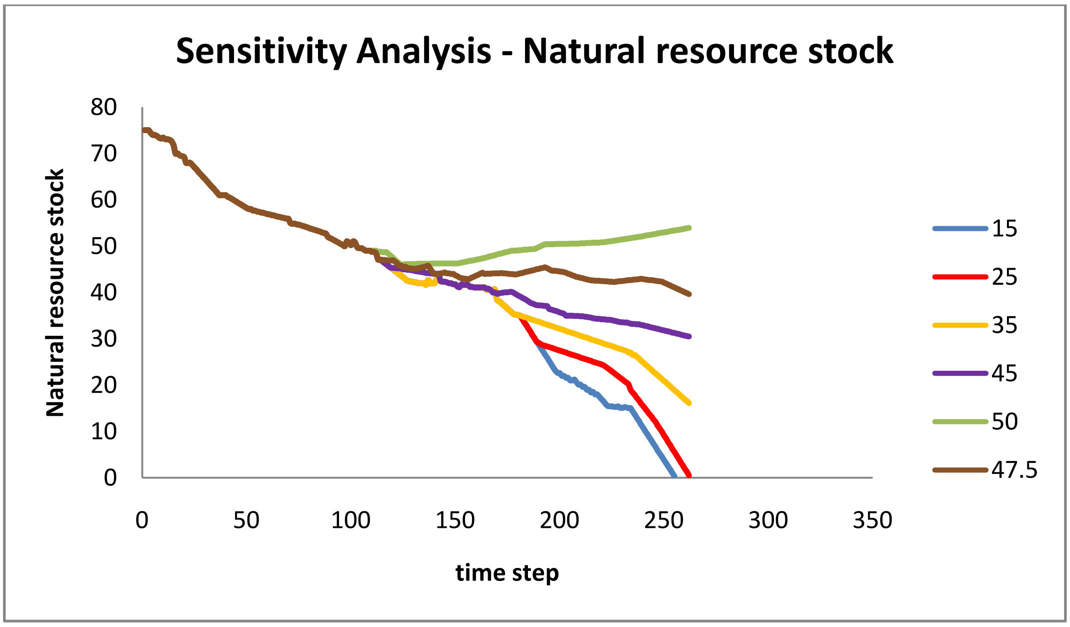

Fig S2. Sensitivity analysis results for the 'Natural resource stock' parameter. The numbers in the legend refer to the different *critical-biomass-stock* values analysed. Note that, contrary to the results in the main paper, no error bands are included here, thus each curve shows the mean value (per time step) from all the runs computed (i.e. 100 runs per *critical-biomass-stock* value).

The sensitivity analysis showed that both 25 and 50 *critical-biomass-stock* values are key thresholds for both ‘Natural resource stock’ and ‘Real GDP growth’ indicators, in comparison to the rest of *critical-biomass-stock* values. Thus, these two values are selected for the analysis performed in the Results section of the main paper. In particular, a *critical-biomass-stock* of 25 shows a sudden decline of both indicators after the time step 150 – including a relative stable (lower) decline from 150 to 225 time steps –, yet still both indicators end up collapsing. In contrast, the closer the *critical-biomass-stock* values get to 50, the more positive (stable) trends are obtained. Thus, a *critical-biomass-stock* of 50 is the lowest value from which a continuous increasing trend (for both ‘Natural resource stock’ and ‘Real GDP growth’ indicators) is obtained.
