# Supplementary material for: It's not the 'what', but the 'how': Exploring the role of debt in natural resource (un)sustainability
Source: PLoS One. 2018 Jul 20;13(7):e0201141. doi: 10.1371/journal.pone.0201141 (PMC6054380; doi:10.1371/journal.pone.0201141)
Supplement: S3 Appendix — Calibration of the ABM, based on a comparative (qualitative) analysis between Keen’s (2009, 2010a) results and our ABM results. (PDF) [file pone.0201141.s003.pdf]

Model calibration

Model calibration was based on performing a comparative analysis between Keen's model (2009, 2010a) results and model outcomes from our ABM. The objective was to test the potential of our model to reproduce similar patterns to those from Keen's models. We used the results obtained from the fractional-reserve (with no government intervention) scenario:

- **Speculation**

  Keen's (2009) Figure 10 shows that a far higher level of debt is accumulated with Ponzi speculation than without it, showing an increase of 100% in 50 years. As a result of this, the increasing debt level causes a complete economic downturn.

  Our results for the 'Speculation rate' indicator show a similar pattern to Keen's rising speculation curve (see Ponzi finance (RHS), Figure 10, Keen (2009)). Note that our results show a drop in speculation during the period 150-200 (time steps), which does not appear in Keen's model. This is because Keen's (2009) model does not integrate an environmental system, including natural resource stocks, in which the economy depends. Thus, his model does not produce endogenous economic (and environmental) collapses, which is the reason why speculation decreases for a short period of time in our model.

- **Debt and GDP**

  Keen's (2010a) Figure 8 shows that, from 1932 until 1943 – i.e. the Great Depression – a collapse of the U.S. national GDP occurred. This collapse was followed by an economic boom, where the private debt to GDP ratio increased in 250% by 2010 compared to 1943.

  Our results for the 'Debt growth rate' indicator show matching patterns to that in Keen's Figure 8 (2010a). Thus, debt stocks increase over time, before an economic crunch hits the system – during the period 1932-1940 in Keen's Figure 8, and during the period 150-200 in our Figure 2 – followed again by an increase in debt due to a (temporal) recovery of the economy. With regard to GDP, our results partially align

with Keen's Figure 8, yet – likewise speculation – our inclusion of environmental constraints to the economy enhance a continuous downturn of GDP after the economic crunch, while Keen's representation of the USA economy shows an increasing GDP trend after the Great Depression (until 2008, when the last economic crisis took place). The fact that our model does not show a continuous growth after the credit crunch could be due to our model not including the relationship between distant coupled SES, including distant economic-environmental links. Thus, real-world economies, such as the U.S. economy, have access to multiple sources of energy and natural resources, i.e. from different countries, at different temporal and spatial scales; whereas our model only simulates the dynamics within one coupled SES. Due to this, the economy in our model has more limited access to natural resources (at least more than Keen's U.S. economy modelled), and this is why our model, in contrast to Keen's model, shows a decreasing trend for GDP after the economic downturn.

- **Monetary capital (firms, households and bank)**

Keen's (2010a) Figure 2 shows the results obtained for bank reserves (i.e. Bank Vault), firms' monetary capital (i.e. Firm Deposit) and households' monetary capital (i.e. Worker Deposit). These show increasing patterns over time, i.e. around 80 and 6 millions of dollars in firm and household deposits, respectively, in 10 years.

Our results show matching patterns for most of the above-noted indicators during the entire simulation. For instance, both household and firm monetary capital in our model increases over time, until the economic downturn takes place. Keen's (2010a) Figure 2 shows, similarly – although with lower oscillations and variability – rising firm and worker (i.e. household) deposits. As previously discussed, the main difference among both models is the inclusion of environmental constraints in our ABM, thus speeding-up the decrease of firm' and households' monetary capital in our model (in contrast to Keen's model, which allows a continuous economic growth). With regard to the bank reserves (i.e. Bank Vault in Keen's model), our ABM and Keen's model show opposite trends. This is because, in our fractional-reserve banking system, the bank has (almost) unlimited bank reserves allocated for credit lending, and the profits gained from the difference between household deposits (losses) and credit interests (gains) is higher than in Keen's model. Furthermore,

Keen’s model is a closed circuit, developed using system dynamics modelling and built under the Circuit Theory (Graziani, 1990). Here, the amount of money circulating in the economy is limited to the initial value set by the modeller.
